# Meta‐Analysis of the Efficacy of Intense Pulsed Light and Pulsed‐Dye Laser Therapy in the Management of Rosacea

**DOI:** 10.1111/jocd.16549

**Published:** 2024-09-06

**Authors:** Qianyu Zhai, Shaohang Cheng, Runying Liu, Jinying Xie, Xiao Han, Zhen Yu

**Affiliations:** ^1^ Department of Dermatology Shenzhen Center for Chronic Disease Control Shenzhen Guangdong China; ^2^ Department of Dermatology Shenzhen People's Hospital Shenzhen Guangdong China

**Keywords:** intense pulsed light, meta‐analysis, pulsed‐dye laser, rosacea, systematic review

## Abstract

**Objective:**

The primary aim of this systematic review and meta‐analysis was to synthesize and compare the clinical efficacy of intense pulsed light (IPL) and pulsed‐dye laser (PDL) therapies for the management of rosacea.

**Methods:**

The literatures were searched in the Web of Science, PubMed, Embase, and Cochrane Library databases to identify relevant studies investigating the use of IPL and PDL for the treatment of rosacea. Screening of the retrieved articles and data extraction were performed as per the pre‐established inclusion and exclusion criteria. The primary outcome measures evaluated in this meta‐analysis included clearance rates, erythema scores, and pain scores.

**Results:**

The meta‐analysis incorporated data from four studies involving a total of 141 participants. The meta‐analysis did not reveal a statistically significant difference between IPL and PDL in the rate of achieving greater than 50% clearance (RR = −0.07, 95% CI: −0.19, 0.05). However, the IPL group demonstrated a significantly higher rate of clearance exceeding 75% compared to the PDL group (RR = −0.13, 95% CI: −0.23, −0.04). The change in erythema index, a key measure of rosacea severity, was similar between the two treatment modalities (SMD = −0.15, 95% CI: −0.55, 0.26). Interestingly, the PDL group reported a notably lower VAS pain score than the IPL group (SMD = 1.54, 95% CI: 0.08, 3.00).

**Conclusion:**

Either PDL or IPL appears to be effective modalities for the management of rosacea. IPL exhibits a slight advantage in achieving a higher rate of substantial (>75%) clearance, while PDL may be preferable for patients with lower tolerance for post‐treatment discomfort. However, the existing literature directly comparing these two laser/light‐based therapies is limited, warranting further well‐designed, large‐scale studies to establish the optimal treatment algorithm for this chronic inflammatory skin condition.

## 1 Introduction

Rosacea is a common chronic inflammatory and vascular skin condition that primarily affects the face, characterized by erythema, flushing, papules, pustules, and telangiectasia, and is one of the most common dermatological outpatient complaints [1]. Rosacea is a persistent condition with a high tendency for recurrence, affecting approximately 5.46% of the global population, and is a common cosmetic concern [2]. Various therapies have been proposed to treat this cosmetic issue, including topical creams, lasers, and light‐based devices, with varying results [3]. Alpha‐adrenergic agonist topical gels have been used to treat erythema and facial telangiectasia, but the effects typically last no more than 12 h and do not improve larger caliber telangiectasia [4]. In recent years, the use of energy‐based and laser/light therapies has significantly increased, as they can provide more durable cosmetic outcomes [5, 6].

Intense pulsed light (IPL) is a broad‐spectrum light source generated by focusing and filtering a high‐intensity light, with a wavelength range of 500–1200 nm, which is a non‐coherent, polychromatic light [7]. Since its FDA approval in 1995, IPL devices have been developed to allow modulation of energy fluence, spectral output, spot size, and pulse duration, and have been recommended for the treatment of various conditions, such as rosacea and photoaging [8]. The broad‐spectrum nature of IPL allows it to be selectively absorbed by the primary chromophores in the skin, such as hemoglobin, melanin, and water, making it effective in improving pigmentation, vascular, and skin quality [9]. Pulsed‐dye laser (PDL) is a laser‐based treatment technology that targets hemoglobin, as hemoglobin has an absorption peak in the 500–600 nm wavelength range [10]. When oxygenated hemoglobin absorbs the laser energy, it generates a localized thermal effect, which is then transferred to the vessel wall, leading to endothelial damage and vessel coagulation, ultimately resulting in vessel occlusion [11] PDL, with its long pulse duration and epidermal cooling technology, has been widely used in the laser treatment of various vascular skin conditions, such as cherry angioma, superficial hemangioma, and telangiectasia [12]. However, the reported efficacy of different treatment modalities varies greatly. Hence, the primary objective of the present study was to rigorously evaluate and compare the clinical efficacy of two prominent light‐based therapies, namely IPL and PDL, in the management of facial rosacea.

## 2 Data and Methods

### 2.1 Search Strategy

The researchers conducted a comprehensive literature search in the Web of Science, PubMed, Embase, and Cochrane Library databases for published articles related to the treatment of rosacea using IPL and PDL. The search terms included: “rosacea”, “PDL”, “IPL”, and “facial telangiectasia.” The search was performed up to March 1, 2024, without language restrictions. Additionally, to further enhance the robustness of the evidence synthesis, the research team conducted a meticulous manual screening of the reference lists from relevant systematic reviews. This additional step was undertaken to identify any potentially eligible studies that may have been overlooked in the initial electronic database searches, thereby ensuring a comprehensive literature review process.

### 2.2 Inclusion and Exclusion Criteria

Inclusion criteria:

1. Patients diagnosed with the various types of facial rosacea.
2. The study compared two treatment methods: PDL and IPL.
3. The study reported outcomes following the interventions, including subjective improvement, clinical efficacy, pain score or erythema index.
4. Study design was a randomized controlled trial (RCT) or cohort study.

Exclusion criteria:

1. Studies involving the concomitant use of drugs or other interventions.
2. The unclear diagnosis of rosacea.
3. Publication types such as comments, letters, or case reports.
4. Duplicate publications or studies utilizing the same data across multiple articles, with only the most comprehensive report included.

### 2.3 Data Extraction and Quality Assessment

Two independent researchers meticulously conducted the literature screening and data extraction processes, strictly adhering to the pre‐established inclusion and exclusion criteria. The extracted data encompassed key details such as publication year, the first author's name, participant demographics (age, sample size, and gender), study location, intervention modalities, and outcome measures of interest.

The methodological quality of the included RCTs was rigorously evaluated using the modified Jadad scale, a well‐validated tool for assessing the risk of bias in clinical studies. Furthermore, the cohort studies were subject to a comprehensive quality assessment utilizing the renowned Newcastle–Ottawa Scale (NOS), a reliable instrument for evaluating the methodological rigor of observational research.

Any inconsistencies in data extraction and quality assessment were resolved through discussion. Discussions that could not be resolved were discussed with the third author to reach a consensus.

### 2.4 Outcome Indicators

The outcome indicators included efficacy and safety indicators. Considering the significant differences in indicators between different studies, a 5‐level scoring system was used to evaluate the efficacy of vascular lesion clearance (none, 0%; poor, 1%–24%; fair, 25%–49%; good, 50%–74%; or excellent, 75%–100%). Visual analogue scoring (VAS) was used to assess safety.

### 2.5 Statistical Analysis

The data analyzed in this meta‐analysis included both categorical and continuous outcome variables. For categorical variables, the risk difference (RR) and its corresponding 95% confidence interval (CI) were used to calculate the pooled effect size. For continuous variables, the standardized mean difference (SMD) and 95% CI were used. Cochran's *Q*‐test was performed to assess the heterogeneity across studies. An *I*_2_ value of ≤25% was considered as low heterogeneity, 25% < *I*_2_ ≤50% as moderate heterogeneity, and *I*_2_ >50% as significant heterogeneity. For studies with low heterogeneity, a fixed‐effects model was employed to calculate the pooled effect size. For moderate or significant heterogeneity, a random‐effects model was used. Funnel plots were generated to evaluate the potential for publication bias. Statistical analyzes were conducted using Stata software, and a two‐sided *p* < 0.05 was considered statistically significant, excluding the heterogeneity test.

## 3 Results

### 3.1 Literature Screening Process

**FIGURE 1:**
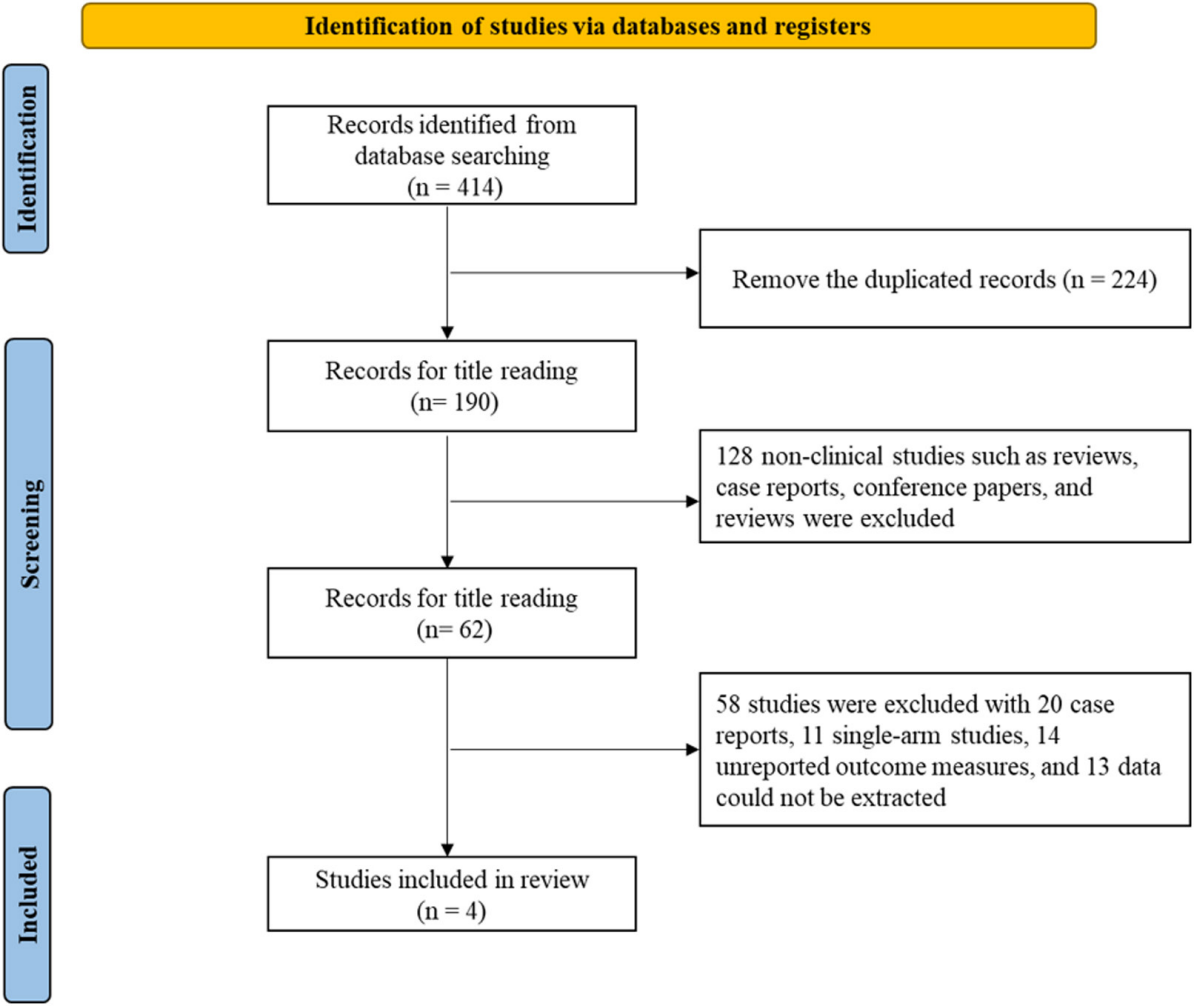
Literature screening flow chart.

A total of 414 articles were retrieved from the databases, and 190 articles were obtained after removing duplicates. After reading the abstracts, 128 non‐clinical studies, including reviews, case reports, conference papers, and commentaries, were excluded. Of the remaining 62 articles, 20 case reports, 11 single‐arm studies, 14 articles without reported outcome indicators, and 13 articles without extractable data were excluded. Finally, four articles [13, 14, 15, 16] that met the eligibility criteria were included. The literature screening process is shown in Figure 1.

### 3.2 Characteristics of Included Studies

Of the four included studies, three [14, 15, 16] were prospective split‐face studies (with different interventions on the left and right sides), and one [13] was a retrospective study. A total of 141 patients were included, and all studies used 595 nm PDL and IPL for treatment. The quality of the included RCT and cohort studies was evaluated using the modified Jadad scale and the NOS, respectively, and the quality was generally high. The basic information of the included studies is shown in Table 1.

**TABLE 1:** Basic information of the included literature.

Study	Years	Design	Patients	*N*	Age (years old)	Intervention	Quality scores
Gao et al. [13]	2019	Retrospective analysis	Facial telangiectasia	38	38.24 ± 0.32	595 nm PDL	6^a^
				39	35.03 ± 11.89	530–650 nm and 900–1200 nm IPL	5^b^
Kim et al. [14]	2019	Randomized, split‐face trial	Facial rosacea	9	40.33 ± 13.13	595‐nm PDL vs. short‐pulsed IPL	6^b^
Nymann et al. [15]	2010	Randomized, split‐face trial	Facial telangiectasias	39	54 (42–63)	595 nm PDL vs. 500–1200 nm IPL	7^b^
Tanghetti [16]	2012	Randomized, split‐face trial	Facial telangiectasias	16	63 (35–85)	595 nm PDL vs. IPL	

^a^ NOS scale.

^b^ Improved Jadad scale.

### 3.3 Efficacy Analysis

#### 3.3.1 Clearance Rate >50%

**FIGURE 2:**
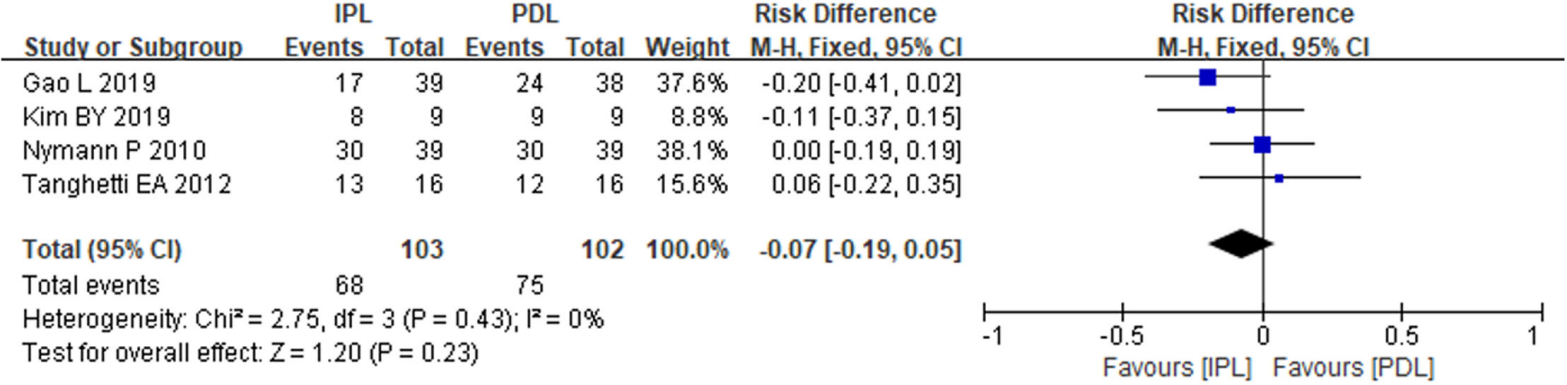
The forest plot of incidence with clearance rate >50%.

The meta‐analysis of the four included studies revealed no significant heterogeneity (*I*_2_ = 0%, *p* = 0.43) in the outcome of clearance rate greater than 50%. A fixed‐effects model was employed for the data synthesis. The pooled results indicated that the incidence of clearance rate exceeding 50% was up to 100% in the PDL group and 88.89% in the IPL group. No statistically significant difference was observed between the two treatment modalities in this outcome (RR = −0.07, 95% CI: −0.19, 0.05) (Figure 2).

#### 3.3.2 Clearance Rate >75%

**FIGURE 3:**
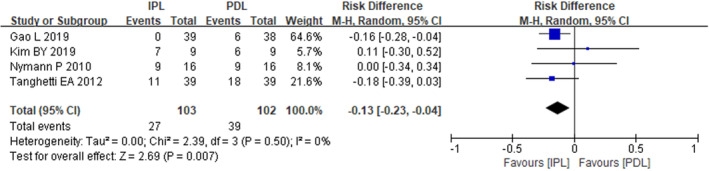
The forest plot of incidence with clearance rate >75%.

All four studies reported the outcome of clearance rate greater than 75%. The heterogeneity assessment showed no significant variability across the studies (*I*_2_ = 0%, *p* = 0.50), and a fixed‐effects model was utilized. The pooled analysis demonstrated that the incidence of clearance rate exceeding 75% was 66.67% in the PDL group and 77.78% in the IPL group. The results indicated a significantly higher clearance rate above 75% in the IPL group compared to the PDL group (RR = −0.13, 95% CI: −0.23, −0.04) (Figure 3).

#### 3.3.3 Erythema Index Assessment

**FIGURE 4:**
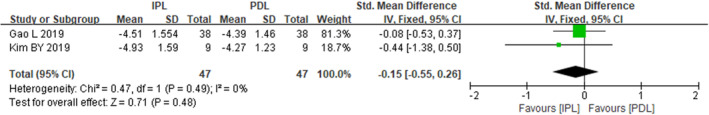
The forest plot of erythema index change.

Two of the included studies reported the erythema index before and after the respective treatment interventions. The meta‐analysis of these studies revealed no significant heterogeneity (*I*_2_ = 0%, *p* = 0.49), allowing for the application of a fixed‐effects model. The pooled results demonstrated no statistically significant difference in the change of erythema index between the IPL and PDL groups (SMD = −0.15, 95% CI: −0.55, 0.26) (Figure 4).

### 3.4 Pain Analysis

**FIGURE 5:**
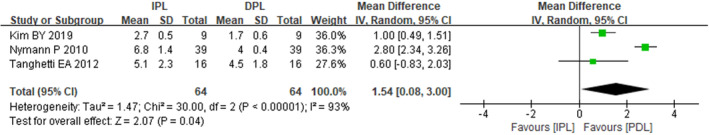
The forest plot of VAS score after treatment.

Three of the four included studies provided data on the VAS pain scores following the treatment procedures. Significant heterogeneity was detected among these studies (*I*_2_ = 93%, *p* < 0.001), necessitating the use of a random‐effects model for the meta‐analysis. The pooled results indicated that the VAS pain scores were significantly lower in the PDL group compared to the IPL group (SMD = 1.54, 95% CI: 0.08, 3.00) (SMD = 1.54, 95% CI: 0.08, 3.00) (Figure 5).

### 3.5 Publication Bias Analysis

**FIGURE 6:**
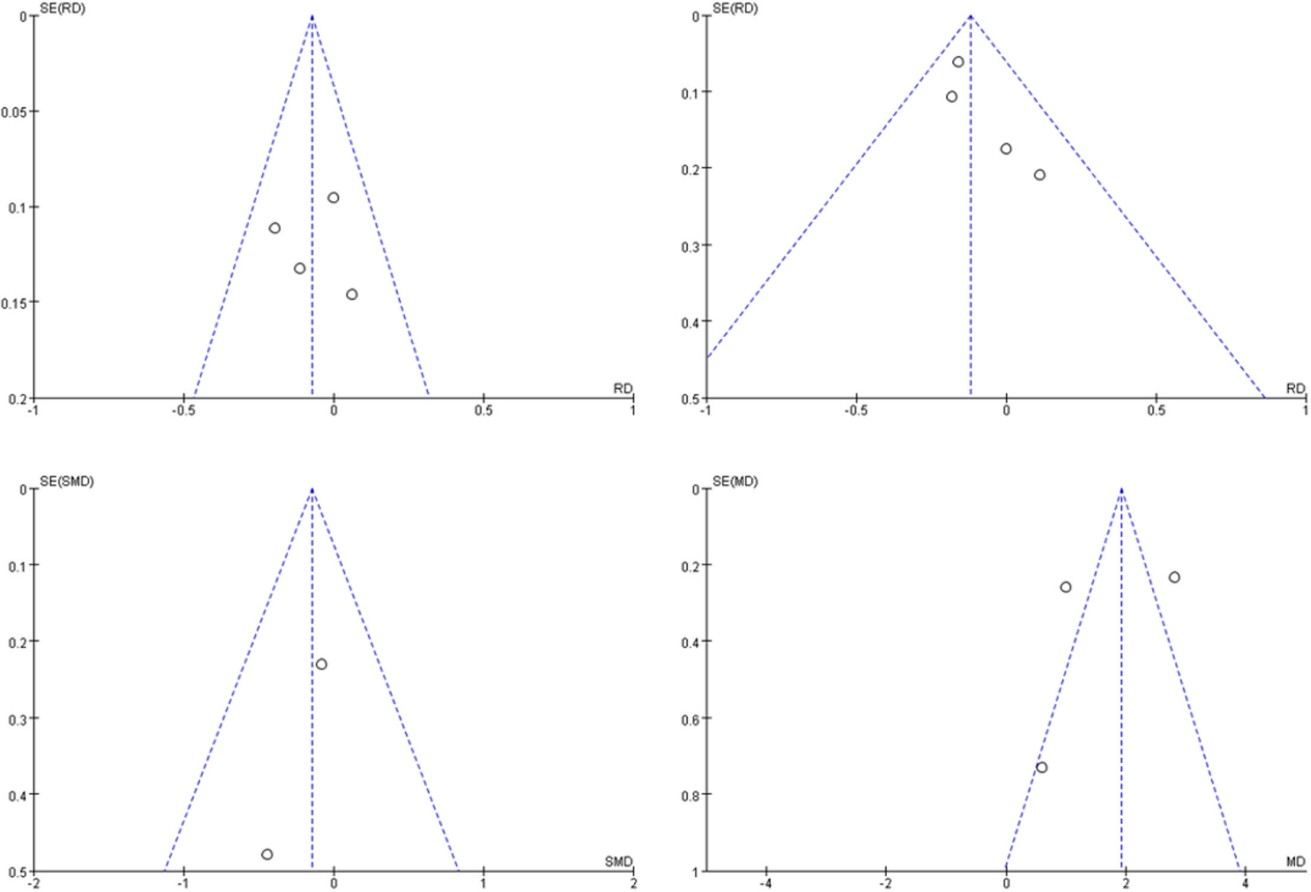
The funnel plot of publication bias.

The funnel plot was used to evaluate the publication bias, as shown in Figure 6. The studies on clearance rate >50%, clearance rate >75%, and erythema index were within the contour, while only one study on VAS score was within the contour. Considering the small number of included studies, there was a certain degree of publication bias (Figure 6).

## 4 Discussion

The findings of this comprehensive systematic review and meta‐analysis suggest that both IPL and PDL demonstrate robust efficacy and favorable safety profiles in the treatment of rosacea. The underlying mechanisms of action for laser and light‐based therapies are related to the reduction of Propionibacterium acnes levels and modulation of sebaceous gland activity. Propionibacterium acnes typically produces porphyrins during its metabolic and proliferative processes, which, when exposed to visible light, can generate highly reactive singlet oxygen species that selectively eliminate the bacteria [17]. The upper dermal sebaceous glands are believed to be heat‐sensitive, and targeted heating of the sebaceous glands can lead to a reduction in their size and decreased sebum production [18].

Rosacea is a common chronic inflammatory cutaneous condition characterized by transient central facial erythema, which may persist due to recurrent episodes, and can also be a concomitant symptom of other systemic dermatological disorders, such as systemic lupus erythematosus, dermatomyositis, drug eruptions, or disseminated bacterial infections [19]. In recent years, energy‐based and laser/light therapies have gained significant prominence in dermatological practice, including PDL, IPL, and others. These treatment modalities are non‐invasive, with rapid onset of action and relatively few adverse effects [20].

PDL utilizes organic dye molecules dissolved in a liquid solvent as the lasing medium, generating short, high‐intensity light pulses using pulsed techniques, targeting and disrupting blood vessels without causing collateral damage to the surrounding skin [21]. PDL has a specific wavelength of 595 nm, which penetrates the skin's surface and is selectively absorbed by oxygenated hemoglobin and hemoglobin in the blood vessels, allowing the surrounding tissue to remain intact [22]. PDL also has an inhibitory effect on vascular endothelial cell proliferation and anti‐inflammatory properties, making it a preferred treatment for vascular skin conditions [11]. Studies have demonstrated that PDL is a safe and effective treatment for rosacea and its telangiectatic component, but it may be less effective in clearing larger areas of erythema [23, 24].

In contrast, IPL is a non‐coherent light source that can simultaneously produce various wavelengths of visible and near‐infrared light. By using appropriate filters, the emitted wavelengths can be tailored to target the size and depth distribution of the desired tissue structures. Studies have shown that, compared to the control group, IPL significantly improved the efficacy rate in the treatment of telangiectasia in late‐stage rosacea, with a lower recurrence rate at 2‐year follow‐up (8.41% vs. 48.33%) [25].

This meta‐analysis indicates that, compared to PDL, IPL has a greater advantage in achieving a clearance rate of >75%, but it is also more painful. Currently, there are more studies on the application of PDL and IPL, and there is limited literature directly comparing the two, with some controversies. Analyzing the reasons, IPL has a wider wavelength range, covering more than one absorption peak of hemoglobin, and has larger spot sizes and broader wavebands, allowing it to effectively treat lesions of various sizes and depths [26]. Skin cooling is a crucial issue in reducing treatment‐related pain and minimizing non‐specific thermal injury and post‐procedural adverse events [27]. PDL is equipped with a dynamic cooling system, which can protect the epidermis and upper dermis from high‐energy thermal damage, providing higher safety [28].

It is worth noting that this meta‐analysis only included four studies with a total of 141 participants, which is a relatively small sample size. Additionally, the settings for PDL and IPL were customized based on the patients' conditions, leading to some heterogeneity among the studies.

In conclusion, both PDL and IPL have shown robust efficacy in the treatment of rosacea. IPL has a certain advantage in improving the high clearance rate (>75%), while PDL has an advantage in reducing post‐treatment pain. However, there is limited research directly comparing the two modalities, necessitating further well‐designed, large‐scale studies to establish the optimal treatment algorithm for rosacea.

## Author Contributions

Qianyu Zhai was the guarantor of integrity of the entire study, carried out study concepts & design, helped to literature research and manuscript editing & review; Shaohang Cheng and Runying Liu contributed to clinical studies, data acquisition; Jinying Xie, Xiao Han and Zhen Yu helped to data & statistical analysis.

## Ethics Statement

All procedures performed in studies involving human participants were in accordance with the 1964 Helsinki declaration and its later amendments or comparable ethical standards. This study is approved by the Ethics Committee of Shenzhen Center for Chronic Disease Control.

## Consent

This meta‐analysis was approved by the institutional review board, the need for informed patient consent for inclusion was waived.

## Conflicts of Interest

The authors declare no conflicts of interest.

## Data Availability

The simulation experiment data used to support the findings of this study are available from the corresponding author upon request.
